# Cardioplegia Strategies in Aortic Stenosis: A Focused Review of Current Evidence for Surgical Considerations

**DOI:** 10.31083/RCM52775

**Published:** 2026-10-14

**Authors:** Minji Ho, Benjamin Chapman, Gianluca Lucchese, Paolo Bosco

**Affiliations:** ^1^Department of Cardiac Surgery, St Thomas’ Hospital, SE1 7EH London, UK; ^2^Department of Physiology, Anatomy, and Genetics, University of Oxford, OX1 3PT Oxford, UK

**Keywords:** aortic valve stenosis, heart arrest, induced, cardioplegic solutions, heart valve prosthesis implantation, hypertrophy, left ventricular, myocardial reperfusion injury, coronary circulation, hypothermia, induced

## Abstract

Severe aortic stenosis (AS) exposes the hypertrophied myocardium to cardioplegic arrest in the setting of increased left ventricle (LV) mass, interstitial fibrosis, impaired relaxation, coronary microvascular dysfunction, and reduced perfusion reserve. These abnormalities may increase susceptibility to ischemic injury and complicate homogeneous cardioplegic delivery. This focused narrative review synthesizes physiological and clinical evidence on cardioplegia composition, temperature, delivery route, dosing, redosing, and myocardial protection during prolonged aortic cross-clamping in adults undergoing surgical aortic valve replacement. AS-specific comparative studies remain limited, generally small, and frequently reliant on biochemical markers; meanwhile, larger observational cohorts and contemporary randomized aortic valve replacement (AVR) trials also vary substantially in patient selection and myocardial protection protocols. Although some studies have associated cold blood cardioplegia with lower biomarker release than cold crystalloid cardioplegia, and cold maintenance strategies with less biochemical injury than warm cardioplegia, no formulation, temperature, or delivery route has demonstrated consistent superiority in clinically important outcomes. Limited historical evidence also suggests that body weight-based dosing may inadequately reflect myocardial requirements in marked LV hypertrophy, but no validated LV mass-based regimen currently exists. Therefore, cardioplegia in severe AS should be considered within a comprehensive myocardial protection protocol incorporating composition, temperature, dose, delivery route, redosing strategy, myocardial phenotype, associated valve or coronary disease, and anticipated cross-clamp duration. Prospective AS-specific studies using standardized protocols, LV mass stratification, and clinically meaningful outcomes are required.

## 1. Introduction

Aortic stenosis (AS) is among the most prevalent valvular heart diseases in high-income countries, and its clinical importance is increasing as populations age [1,2]. Calcific degeneration accounts for most disease in older adults, whereas bicuspid valve disease commonly produces clinically significant stenosis at a younger age and may be accompanied by aortic pathology [3].

When intervention is indicated, definitive treatment consists of transcatheter or surgical aortic valve replacement [2]. Despite the expansion of transcatheter treatment, surgical aortic valve replacement (AVR) remains appropriate or necessary in selected patients, including those with bicuspid valve disease, associated aortopathy, concomitant coronary or multivalvular disease, infective pathology, or other structural abnormalities requiring operative correction [2]. These patients may undergo isolated AVR or more complex combined procedures during which myocardial protection must be maintained throughout aortic cross-clamping and cardioplegic arrest.

The myocardium exposed to cardioplegia in severe AS differs from that encountered in many other cardiac procedures. Chronic pressure overload produces left ventricular hypertrophy, impaired relaxation, interstitial fibrosis, coronary microvascular dysfunction, and reduced perfusion reserve [4,5,6]. These abnormalities may increase susceptibility to ischaemic injury and negatively affect cardioplegic distribution. Increased left ventricle (LV) mass is particularly relevant as empirical body-weight-based dosing commonly used in practice may not adequately reflect the true amount of myocardium requiring perfusion [7].

Cardioplegic practice varies substantially in solution composition, temperature, delivery route, administered volume, and redosing interval. Evidence specific to severe AS and the hypertrophied LV remains limited. Available studies vary in population, cardioplegia composition, temperature, route, dose, and redosing protocol and frequently alter several of these variables simultaneously. The literature therefore does not support a single preferred strategy or clearly establish how myocardial protection should be adapted to LV mass, ventricular function, coronary anatomy, or anticipated cross-clamp duration.

This focused narrative review critically evaluates cardioplegic myocardial protection in adults undergoing surgical AVR for AS. It examines the haemodynamic and microvascular consequences of AS, the characteristics of commonly used cardioplegic strategies, the effects of temperature and delivery route, the relationship between cardioplegia dose and LV mass, and the maintenance of protection during prolonged cross-clamping. The review aims to identify myocardial and procedural factors that may inform cardioplegic planning and to define priorities for future clinical investigation.

This review was conducted as a focused narrative synthesis. Searches were performed in PubMed, Embase, and the Cochrane Library and updated in July 2026 using combinations of the terms “aortic stenosis”, “aortic valve replacement”, “cardioplegia”, “myocardial protection”, “left ventricular hypertrophy”, “Del Nido”, “Custodiol”, “HTK”, “blood cardioplegia”, “crystalloid cardioplegia”, “antegrade cardioplegia”, and “retrograde cardioplegia”. Comparative human studies involving AS or AVR were prioritised. Studies from mixed cardiac surgery populations, other valve procedures, and preclinical models were considered where they addressed questions for which AS-specific evidence was unavailable. Older studies were retained where foundational or where no contemporary alternative existed. No formal inclusion or exclusion criteria, protocol registration, risk-of-bias assessment, or quantitative synthesis were applied. The review should therefore be interpreted as a focused narrative review rather than a systematic review or meta-analysis.

## 2. Overview of Aortic Stenosis

AS is characterised by progressive obstruction of left ventricular outflow, producing a chronic pressure load that drives compensatory concentric hypertrophy. This response initially reduces systolic wall stress and preserves cardiac output, but becomes maladaptive as disease progresses. Increasing LV mass is accompanied by impaired relaxation, elevated filling pressures, extracellular matrix expansion, and interstitial fibrosis, ultimately reducing ventricular compliance and predisposing to systolic dysfunction and heart failure [3,4,8]. Clinical presentation can therefore reflect both the severity of valvular obstruction and the myocardial response to sustained afterload. The classical symptoms of angina, syncope, and dyspnoea usually emerge after a prolonged asymptomatic phase and indicate advanced haemodynamic consequences of disease [8].

AS most commonly results from progressive calcific degeneration, although bicuspid valve disease may produce clinically significant stenosis at a younger age [3]. Disease progression varies according to valve anatomy, calcific burden, haemodynamic severity, and associated cardiovascular disease. The development of symptoms or LV dysfunction marks a transition to clinically advanced disease for which valve intervention should be considered.

The pattern and extent of ventricular remodelling vary considerably between patients. Some develop marked concentric hypertrophy with preserved ejection fraction, whereas others progress to ventricular dilatation, impaired systolic function, and decompensation. Diffuse myocardial fibrosis may be present despite preserved ejection fraction and is associated with impaired diastolic function, greater symptom burden, and adverse clinical outcomes [4]. Conventional assessment of systolic function alone may therefore underestimate the severity of myocardial involvement. Measurements of LV wall thickness, mass, dimensions, strain, diastolic function, and fibrosis provide additional information regarding the myocardial substrate exposed to cardioplegic arrest.

Echocardiography remains the principal investigation for diagnosis and severity assessment. High-gradient severe AS is conventionally characterised by an aortic valve area <1.0 cm^2^, a mean transvalvular gradient >40 mmHg, or a peak transvalvular velocity >4 m/s [2]. Discordant measurements and low-flow states require integrated interpretation alongside flow status, LV function, valve morphology, and measurement quality. Assessment of LV hypertrophy is particularly relevant to myocardial protection because patients with similar valve gradients may have substantially different myocardial mass, which may influence the volume of cardioplegia required for adequate myocardial exposure [7].

At present, there is no medical therapy that reverses established valvular obstruction. Aortic valve replacement, either surgical or transcatheter, remains the definitive treatment for severe disease [2]. Transcatheter aortic valve implantation (TAVI) has become an important treatment across a broad range of surgical-risk groups, but open AVR remains necessary for many patients, including those with bicuspid valve disease, aortopathy, concomitant coronary artery disease, multivalve pathology, infective endocarditis, or other structural abnormalities requiring surgical correction. These patients may undergo prolonged or complex procedures and frequently have substantial LV hypertrophy, making the adequacy of cardioplegic arrest and myocardial protection directly relevant to operative outcome.

The severity of valvular obstruction alone does not fully define the myocardial risk encountered during surgery. LV mass, fibrosis, impaired relaxation, systolic function, and associated coronary disease collectively determine the myocardial substrate exposed to cardioplegic arrest. The haemodynamic and microvascular consequences of this remodelling are considered in the following section.

## 3. Haemodynamics in AS

Severe AS imposes a sustained pressure load on the LV and produces a distinctive myocardial haemodynamic environment. Concentric hypertrophy initially compensates for increased systolic wall stress, but the increase in myocardial mass is not matched proportionately by expansion of the coronary microcirculation. The resulting mismatch between myocardial demand and vascular supply contributes to coronary microvascular dysfunction, impaired perfusion reserve, and increased vulnerability to ischaemia, even in the absence of obstructive epicardial coronary disease [6,9].

Cardiac magnetic resonance imaging has demonstrated reduced myocardial perfusion reserve and impaired oxygenation during vasodilator stress in patients with severe AS [5]. These abnormalities are accompanied by impaired myocardial energetics and subclinical LV dysfunction and may improve following relief of valvular obstruction, supporting a direct relationship with AS rather than concomitant coronary disease alone [5]. The myocardium may remain adequately oxygenated at rest, but its capacity to increase perfusion in response to physiological or pharmacological stress is limited. Similar dissociation between resting perfusion and stress-induced oxygenation abnormalities has been demonstrated in other forms of coronary disease and emphasises that preserved resting flow does not imply normal myocardial reserve [10,11].

Several mechanisms contribute to this impaired reserve. Capillary and arteriolar growth do not keep pace with increasing myocardial mass, resulting in reduced flow per gram of myocardium in hypertrophied compared with non-hypertrophied ventricles [9]. Increased intracavitary and intramyocardial pressures compress the coronary microcirculation, while prolonged LV ejection and impaired diastolic relaxation reduce the effectiveness of the diastolic interval during which most left-coronary flow occurs. Interstitial fibrosis and structural remodelling may further impair regional perfusion and diffusion [4,12]. Increased resistance has been identified in both coronary circulations, suggesting that these abnormalities affect the myocardium globally rather than being confined to a single vascular territory [9].

Coronary flow reserve is consequently reduced in severe AS. Echocardiographic studies have demonstrated impaired hyperaemic flow relative to LV mass, with minimal systolic contribution and an inadequate increase in peak diastolic velocity [13]. Reverse systolic coronary flow may also occur as a consequence of intramyocardial compression and altered ventriculo-coronary interaction, further reducing net systolic perfusion. These abnormalities may be present despite angiographically normal coronary arteries and demonstrate improvement after valve replacement, indicating that they arise predominantly from the haemodynamic and structural effects of AS [14,15].

These physiological abnormalities have important implications for myocardial protection. Cardioplegia must be distributed through a coronary circulation supplying a larger myocardial mass with impaired reserve and potentially heterogeneous microvascular conductance [6,9]. Rapid electrical arrest and myocardial cooling do not necessarily confirm homogeneous or quantitatively adequate cardioplegic distribution throughout the hypertrophied ventricle [7].

The haemodynamic conditions during cardioplegic arrest differ substantially from those of the beating heart. Cardiopulmonary bypass decompresses the LV, while electromechanical arrest removes the transvalvular pressure gradient, systolic contraction, and much of the dynamic extravascular compression affecting physiological coronary flow [16,17]. However, increased myocardial mass, fibrosis, mismatch between myocardial mass and microvascular supply, and altered resistance-vessel function remain relevant to cardioplegic distribution [4,6,9]. Physiological measurements of coronary reserve therefore provide mechanistic context rather than a direct model of flow during arrest.

The interaction between AS and concomitant epicardial coronary disease creates an additional challenge. Obstructive coronary lesions may produce regional maldistribution during antegrade cardioplegia, superimposed on the diffuse coronary microvascular dysfunction associated with hypertrophy [6,16]. Patients undergoing combined AVR and coronary artery bypass grafting may also experience longer cross-clamp times and require different delivery strategies from those undergoing isolated valve replacement [18].

Severe AS therefore produces a myocardium with increased mass, impaired perfusion reserve, and functional and structural abnormalities of the coronary microcirculation [5,9]. These features provide a physiological rationale for considering cardioplegia dose, temperature, delivery route, and redosing specifically in relation to the hypertrophied ventricle, while recognising that physiological coronary-flow measurements cannot determine the optimal cardioplegic strategy directly.

## 4. Cardioplegic Strategies

Surgical AVR may be performed as an isolated procedure or alongside coronary, valvular, or aortic surgery and generally requires aortic cross-clamping and cardioplegic arrest to provide a still, bloodless operative field [2]. Cardioplegia produces rapid electromechanical arrest while reducing myocardial energy consumption and limiting ischaemia-reperfusion injury during the cross-clamp period [18,19].

Cardioplegic strategies vary in composition, mechanism of arrest, temperature, delivery route, volume, and redosing interval. These variables are interdependent, and named formulations are commonly modified according to institutional practice. Comparative studies therefore usually assess complete myocardial-protection protocols rather than composition alone.

Most cardioplegic solutions induce depolarised arrest through a high extracellular potassium concentration. Partial membrane depolarisation inactivates fast sodium channels and prevents action-potential propagation. Hypothermia, buffering, osmotic control, and limitation of intracellular calcium accumulation provide additional protection [17,18,19]. Prolonged depolarisation may nevertheless promote sodium and calcium loading, which has encouraged the use of membrane-stabilising and calcium-modulating additives.

Blood cardioplegia combines oxygenated blood from the cardiopulmonary bypass circuit with a hyperkalaemic crystalloid component. It optimises oxygen-carrying capacity, physiological buffering, and reduced crystalloid loading, but its distribution is influenced by haematocrit, temperature, perfusion pressure, and the increased viscosity of cooled blood. Buckberg and Calafiore protocols are widely used examples, although their composition and administration vary substantially [16].

Conventional extracellular crystalloid solutions, including St Thomas’ formulations, contain potassium together with electrolytes and buffering constituents intended to preserve ionic and acid-base homeostasis [18]. Their low viscosity and standardised composition facilitate delivery, but they lack intrinsic oxygen-carrying capacity and may contribute to haemodilution, electrolyte disturbance, and tissue oedema when administered repeatedly. Both blood and crystalloid solutions may be used in multidose protocols, permitting supplementation during prolonged cross-clamping at the cost of operative interruption.

Del Nido cardioplegia was developed for paediatric cardiac surgery and is now widely used in adult practice. Conventional Del Nido cardioplegia is crystalloid-dominant and usually contains a smaller blood component, although bloodless modifications are also used. Potassium induces depolarised arrest, lidocaine limits sodium influx, magnesium antagonises calcium entry, and bicarbonate and mannitol provide buffering and osmotic protection [20]. Its prolonged duration of action may reduce redosing and operative interruption, but adult formulations and redosing thresholds vary, and protection during unexpectedly prolonged cross-clamping or in marked LV hypertrophy remains uncertain. Contemporary comparative evidence is considered in Section 5 [21,22].

Custodiol, or histidine–tryptophan–ketoglutarate (HTK) solution, is a low-sodium, low-calcium intracellular-type crystalloid formulation. Its ionic composition suppresses membrane excitability, while histidine provides buffering, ketoglutarate acts as a metabolic substrate, and tryptophan and mannitol contribute membrane-stabilising and osmotic effects [23,24]. Custodiol is delivered as a comparatively large-volume infusion and is intended to provide prolonged protection without frequent redosing. Its large crystalloid volume may contribute to haemodilution, while its very low sodium concentration is recognised to produce acute iso-osmotic hyponatraemia [16,24]. Protection also depends on adequate initial delivery, myocardial temperature, homogeneous distribution, and the myocardial mass requiring perfusion.

Cardioplegia may be delivered cold, tepid, or warm. Hypothermia reduces myocardial oxygen demand and prolongs tolerance of ischaemia. Warm or tepid cardioplegia avoids profound myocardial hypothermia but requires continuous and homogeneous delivery as interruption or maldistribution may produce anaerobic metabolism and warm ischaemic injury [16]. Temperature must therefore be interpreted alongside composition, dose, route, and redosing rather than as an isolated variable.

A terminal dose of warm cardioplegia, or “hot shot”, may be administered immediately before release of the aortic cross-clamp to replenish metabolic substrates and provide controlled reperfusion [25,26]. This should be distinguished from warm maintenance cardioplegia throughout the cross-clamp period.

Cardioplegia may be administered antegrade through the aortic root, directly into the coronary ostia after aortotomy, retrograde through the coronary sinus, or using a combined approach [18,27]. Antegrade delivery is commonly used in isolated AS, though significant aortic regurgitation may compromise root administration by allowing cardioplegia to enter and distend the LV.

Retrograde coronary-sinus cardioplegia was developed as an alternative route where antegrade delivery was difficult or potentially incomplete [28]. Its effectiveness depends on coronary venous anatomy, catheter position, flow, and perfusion pressure. Experimental evidence indicates that right-ventricular microvascular perfusion may be incomplete during retrograde-only delivery [16]. Combined antegrade and retrograde delivery may therefore be considered where aortic regurgitation, coronary disease, difficult ostial access, or procedural complexity raises concern about maldistribution. AS-specific comparative evidence has not demonstrated consistent superiority of retrograde cardioplegia [27].

Single-dose protocols commonly estimate volume from body size or assumed cardiac mass, whereas multidose strategies permit supplementation according to elapsed time, recurrent electrical activity, myocardial temperature, and operative circumstances [29]. Cardioplegic adequacy is assessed indirectly using postoperative cardiac troponin and creatine kinase-myocardial band (CK-MB) and intraoperative indicators including persistence of arrest, myocardial temperature, rhythm recovery, and the need for defibrillation or mechanical support [30,31]. None is specific to cardioplegia failure.

Cardioplegic strategies should therefore be considered as complete protocols incorporating composition, temperature, volume, route, redosing, and anticipated cross-clamp duration. In severe AS, the principal uncertainties concern adequate dosing for increased LV mass, homogeneous distribution, and the maintenance of protection during prolonged arrest.

## 5. Challenges of Cardioplegia in AS

Severe AS presents specific challenges for myocardial protection because increased LV mass, coronary microvascular dysfunction, and impaired perfusion reserve may reduce the homogeneity and duration of cardioplegic protection [6]. The principal uncertainties concern temperature, dose, composition, delivery route, and prolonged cross-clamping. Selected pivotal clinical studies are summarised in Table 1 (Ref. [7,22,32,33,34]).

**Table 1.** **Selected clinical studies informing cardioplegic myocardial protection relevant to aortic stenosis**.

Study and population	Comparison	Main findings	Interpretation
Matsuda et al., 1986 [7]: 28 patients with chronic aortic valve disease and LV hypertrophy undergoing AVR	Initial cold crystalloid cardioplegia dose relative to LV mass	Dose per gram of LV mass was inversely associated with peak postoperative CK-MB; doses ≥2.5 mL/g were associated with lower enzyme release.	Supports consideration of LV mass when determining dose. The small historical study used a specific crystalloid protocol, and its threshold should not be transferred directly to contemporary strategies.
Ascione et al., 2002 [32]: randomised study of 35 patients with LV hypertrophy secondary to AS undergoing AVR	Intermittent antegrade cold versus warm blood cardioplegia	Cold cardioplegia was associated with less metabolic stress and lower troponin I release at 1, 24 and 48 hours.	Directly examines temperature in the hypertrophied AS heart, but is limited by its small sample and reliance on metabolic and biochemical outcomes.
Braathen and Tønnessen, 2010 [33]: randomised study of 80 patients undergoing isolated AVR for AS without significant coronary or other valvular disease	Intermittent antegrade cold blood versus cold crystalloid cardioplegia	Peak CK-MB and troponin T were approximately twofold higher with crystalloid cardioplegia.	Directly applicable to isolated AS and avoids confounding by temperature. However, it was modest in size and not powered to establish differences in major clinical outcomes.
Hoyer et al., 2017 [34]: 7263 isolated AVR procedures identified, with 825 propensity-matched pairs	Custodiol/HTK versus intermittent cold Calafiore blood cardioplegia	Operative mortality, postoperative complications and long-term survival were similar. A subgroup analysis suggested improved survival with blood cardioplegia among patients with LVEF ≤30%.	Large and clinically informative, but retrospective and susceptible to treatment-selection, temporal and institutional confounding. It included mixed aortic valve pathology rather than AS alone.
Tauron-Ferrer et al., 2025 [22]: two-centre randomised trial of 311 adults undergoing first-time isolated AVR	Buckberg versus Del Nido cardioplegia protocols	Peak CK and high-sensitivity troponin T were similar. Del Nido was associated with more spontaneous rhythm recovery and less ventricular fibrillation requiring defibrillation.	Supports comparable myocardial protection with Del Nido. The population included stenosis, regurgitation and mixed disease; delivery route and redosing also differed between protocols, and outcomes were not stratified by LV mass.

Studies were selected to represent the principal questions of myocardial-mass-informed dosing, temperature, blood versus crystalloid cardioplegia, prolonged single-dose protection and contemporary Del Nido use. Differences in population and complete cardioplegia protocols preclude direct ranking of strategies. LV, left ventricle; AVR, aortic valve replacement; CK-MB, creatine kinase-myocardial band; AS, aortic stenosis; HTK, histidine–tryptophan–ketoglutarate; LVEF, left ventricular ejection fraction.

Temperature may influence injury in the hypertrophied ventricle. Ascione et al. [32] compared intermittent antegrade cold and warm blood cardioplegia in patients with LV hypertrophy secondary to AS and reported greater troponin I release and anaerobic metabolic stress with warm cardioplegia. Because both groups received blood cardioplegia, the study assesses temperature more directly than comparisons in which composition also differs. Its small sample and reliance on biochemical outcomes nevertheless limit clinical inference.

Nardi et al. [35] reported lower troponin I and CK-MB concentrations with cold crystalloid than warm blood cardioplegia after AVR, without significant differences in major clinical outcomes. Because both temperature and composition differed, the results do not establish crystalloid superiority and are equally compatible with less effective protection from warm maintenance cardioplegia. The available evidence therefore supports caution with warm maintenance strategies more strongly than it favours a particular solution composition. Terminal warm reperfusion represents a distinct intervention.

Marked LV hypertrophy raises concern that an empirical dose based on body size may not reflect the myocardial mass requiring protection. Matsuda et al. [7] found an inverse association between the initial cold crystalloid dose per gram of LV mass and peak postoperative CK-MB, with lower enzyme release among patients receiving at least 2.5 mL/g. The small historical study does not establish a contemporary dosing threshold, but it supports prospective evaluation of LV-mass-informed dosing.

Myocardial cooling cannot confirm complete protection in isolation. Matsuda et al. [7] observed biochemical injury in relatively underdosed patients despite deep myocardial hypothermia. A satisfactory regional temperature may therefore coexist with inadequate total delivery or heterogeneous distribution, while progressive rewarming may reduce protection during prolonged arrest [36].

Increasing crystalloid volume to account for hypertrophy may cause haemodilution, dilutional hyponatraemia, acid-base disturbance, and tissue oedema, particularly with Custodiol-based strategies [16,24,36,37]. The dosing problem therefore involves balancing adequate myocardial exposure against the systemic effects of large-volume administration.

The relationship between composition and outcome remains uncertain. Braathen and Tønnessen randomised 80 patients undergoing isolated AVR for AS to cold blood or cold crystalloid cardioplegia and found lower postoperative troponin T and CK-MB release with blood cardioplegia [33]. The comparison is directly relevant to isolated AS and avoids confounding by temperature, but the modest sample and emphasis on surrogate outcomes prevent definitive clinical conclusions.

Larger observational evidence has not demonstrated consistent superiority of blood cardioplegia. Hoyer et al. [34] compared Custodiol with cold Calafiore blood cardioplegia in isolated AVR. After propensity matching, operative mortality, postoperative complications, and long-term survival were similar. A possible survival advantage with blood cardioplegia among patients with left ventricular ejection fraction (LVEF) ≤30% was hypothesis-generating. Interpretation is limited by non-randomised treatment allocation, a study period exceeding two decades, and institutional and temporal confounding. Other retrospective AVR studies have likewise shown no consistent clinical superiority of blood cardioplegia [38].

The Braathen and Nardi findings are not directly contradictory. Braathen and Tønnessen [33] compared cold blood with cold crystalloid cardioplegia in isolated AS, whereas Nardi et al. [35] compared cold crystalloid with warm blood cardioplegia in a broader AVR population. The studies differed in temperature, patient selection, treatment allocation, concomitant procedures, and biomarker timing. Together, they suggest that temperature and the overall delivery protocol may be at least as important as the blood or crystalloid component alone.

Historical and indirect evidence provides mechanistic context but limited guidance for contemporary AS surgery. Jin et al. [39] reported earlier ventricular functional recovery after cold blood cardioplegia than after warm blood or crystalloid strategies following AVR. Fannelop et al. [40] found superior post-ischaemic function with multidose cold oxygenated blood cardioplegia compared with single-dose Custodiol in a porcine model. Conversely, Custodiol and repetitive cold blood cardioplegia produced comparable protection in mitral valve surgery [41]. Differences in era, species, myocardial substrate, and procedure prevent direct extrapolation to severe calcific AS.

Contemporary studies support the feasibility of prolonged-action formulations but do not identify a superior strategy. Tauron-Ferrer et al. [22] randomised 311 adults undergoing first-time isolated AVR to Buckberg or Del Nido protocols. Biomarker release was similar, while Del Nido was associated with more spontaneous rhythm recovery, less ventricular fibrillation requiring defibrillation, and fewer redosing interruptions. However, the population included stenotic, regurgitant, and mixed valve disease, and outcomes were not stratified by LV mass.

Sohn et al. [21] compared modified bloodless Del Nido cardioplegia with Custodiol in isolated AVR. Mortality was similar, while Del Nido was associated with more spontaneous rhythm recovery, lower early lactate, and less low-cardiac-output syndrome. The retrospective design, temporal confounding, and use of an institution-specific bloodless formulation limit generalisability.

Yücel et al. [42] compared Del Nido and Custodiol in 320 propensity-matched adults undergoing mixed cardiac procedures with cross-clamp times of at least 90 minutes. Del Nido required less cardioplegia and was associated with fewer transfusions and less ventricular fibrillation. In subgroup analyses beyond 180 minutes, Custodiol was associated with lower troponin I and less intra-aortic balloon pump use. These findings suggest that cross-clamp duration may influence relative performance, but the study was retrospective, heterogeneous, and neither AS-specific nor stratified by LV mass.

Delivery route remains another uncertainty. Antegrade cardioplegia is generally appropriate where aortic-root or direct ostial delivery is satisfactory. Retrograde administration may supplement antegrade delivery in the presence of aortic regurgitation, coronary disease, difficult ostial access, or concern about maldistribution [28]. Comparative evidence has not demonstrated consistent superiority of retrograde cardioplegia in AS, supporting its selective use as a complementary rather than routine route [27].

As summarised in Table 1, the evidence remains heterogeneous and does not identify a universally superior cardioplegic strategy for severe AS. Direct AS-specific studies are generally small and biomarker-focused, whereas larger observational cohorts remain vulnerable to treatment-selection bias and institutional variation. Evidence from mixed procedures, other valve populations, and experimental models offers useful context but cannot reliably determine practice in the hypertrophied AS ventricle.

The available literature therefore supports evaluation of the complete myocardial-protection protocol rather than solution composition alone. Marked LV hypertrophy may reduce the reliability of body-weight-based dosing; prolonged procedures require a defined strategy for continued protection; and associated regurgitation or coronary disease may require modification of delivery. These considerations support individualisation according to myocardial phenotype and procedural complexity, but evidence remains insufficient for definitive solution-specific recommendations.

## 6. Future Directions and Additional Considerations

A central unresolved question is whether cardioplegia dosing in severe AS should account directly for LV mass. This is particularly relevant to single-dose strategies, in which the initial volume must achieve homogeneous arrest and protection throughout a hypertrophied ventricle for the intended duration of cross-clamping. Total body weight dosing is convenient but may correlate poorly with myocardial mass in patients with marked concentric hypertrophy. Matsuda et al. [7] found that cardioplegia volume indexed to LV mass was more closely associated with postoperative CK-MB release than body-weight-indexed dosing. Although the numerical threshold identified in this small historical study should not be transferred directly to contemporary blood, Del Nido, or Custodiol protocols, the findings support prospective evaluation of myocardial-mass-informed dosing using preoperative echocardiographic or cardiac magnetic resonance measurements.

Myocardial temperature may document regional cooling but cannot establish adequate dose or homogeneous protection in isolation. Matsuda et al. [7] observed postoperative biochemical injury in relatively underdosed patients despite deep myocardial hypothermia. Future studies should therefore evaluate temperature together with delivered volume, perfusion pressure, distribution, recurrent electrical activity, and redosing, particularly in more complex settings including concomitant ischaemic heart disease.

Increasing crystalloid volume may contribute to haemodilution precipitating dilutional metabolic disturbances, particularly the documented complications of acute iso-osmotic hyponatraemia seen in Custodiol. These effects may be more important in patients with low body mass, renal dysfunction, impaired ventricular function, or prolonged cardiopulmonary bypass. Myocardial protection and systemic metabolic effects should therefore be assessed together rather than assuming that a larger dose is uniformly beneficial.

Future studies should stratify patients according to myocardial phenotype and procedural complexity rather than AS diagnosis alone. Relevant characteristics include LV mass index, wall thickness, systolic and diastolic function, myocardial fibrosis, associated aortic regurgitation, epicardial coronary disease, and anticipated cross-clamp duration. Patients undergoing uncomplicated isolated AVR are unlikely to have the same protection requirements as those undergoing combined coronary, multivalve, redo, or aortic procedures.

Comparative studies should also report the complete myocardial-protection protocol. At minimum, this should include solution composition, blood-to-crystalloid ratio where applicable, initial volume, method of dose indexing, infusion pressure and duration, temperature, delivery route, redosing interval, terminal reperfusion strategy, and total cross-clamp time. Comparisons based only on solution name risk attributing outcomes to composition when they may instead reflect dose, temperature, route, or institutional technique.

Outcome assessment should extend beyond postoperative enzyme release. Troponin and CK-MB remain useful surrogate markers, but they are influenced by multiple perioperative factors and should be interpreted alongside clinically meaningful outcomes [30,31]. Relevant endpoints include ventricular fibrillation and defibrillation after cross-clamp removal, inotropic support, low-cardiac-output syndrome, mechanical circulatory support, perioperative myocardial infarction, renal injury, intensive-care stay, mortality, and postoperative ventricular function. Longer-term studies should also assess LV recovery and reverse remodelling.

The contemporary surgical population must be considered in the context of TAVI. The transcatheter approach avoids aortic cross-clamping and cardioplegic arrest, and both TAVI and surgical AVR are followed by LV mass regression after relief of valvular afterload, although its extent and time course vary between populations and procedures [43]. Greater LV mass regression has been associated with improved outcomes after TAVI and with improved long-term survival after surgical AVR [44,45]. Despite increasing use of TAVI, surgical AVR remains necessary in patients with bicuspid valve disease, aortopathy, concomitant coronary artery disease, multivalve pathology, infective endocarditis, or other structural abnormalities requiring operative correction [2]. These patients may undergo longer and more complex procedures than historical isolated-AVR cohorts, maintaining the clinical importance of optimising cardioplegic protection.

The most informative next step would be a prospective multicentre study of contemporary cardioplegic strategies in patients undergoing surgical AVR for severe AS. Prespecified stratification by LV mass index, ventricular function, and cross-clamp duration would allow assessment of whether myocardial-mass-informed dosing, planned redosing, temperature, or combined delivery improves protection. Standardised reporting of the full cardioplegia protocol and inclusion of both biochemical and clinical endpoints would be essential.

## 7. Conclusions

Severe AS creates a distinctive myocardial substrate for cardioplegic protection. Increased LV mass, interstitial fibrosis, impaired relaxation, coronary microvascular dysfunction, and reduced perfusion reserve may increase vulnerability to ischaemic injury and complicate homogeneous delivery of cardioplegia. These features provide a physiological rationale for adapting myocardial protection to the hypertrophied ventricle.

The available evidence does not identify a universally optimal cardioplegic strategy regarding formulation, temperature, or delivery route. Small AS-specific studies have associated cold blood cardioplegia with lower postoperative biomarker release than cold crystalloid cardioplegia and cold maintenance cardioplegia with less biochemical injury than warm maintenance strategies [32,33]. However, larger observational cohorts and contemporary randomised AVR studies have not demonstrated consistent superiority in clinically important outcomes [22,34]. Cardioplegia should therefore be considered as a complete myocardial-protection protocol incorporating composition, temperature, dose, delivery route, redosing, and anticipated cross-clamp duration rather than by solution name alone.

Body-weight-based dosing may inadequately represent myocardial requirements in patients with marked LV hypertrophy, although current evidence is insufficient to define a validated LV-mass-based dosing strategy [7]. In current practice, myocardial protection should be individualised according to LV mass and function, associated aortic regurgitation or coronary disease, procedural complexity, and the anticipated duration of arrest. Prospective AS-specific studies using standardised protocols and clinically meaningful outcomes are required before definitive solution- or dose-specific recommendations can be made.

## Abbreviations

AS, aortic stenosis; AVR, aortic valve replacement; LV, left ventricle; TAVI, transcatheter aortic valve implantation; HTK, histidine–tryptophan–ketoglutarate; CK-MB, creatine kinase-myocardial band; LVEF, left ventricular ejection fraction.
